# Improving Performance of Tin-Doped-Zinc-Oxide Thin-Film Transistors by Optimizing Channel Structure

**DOI:** 10.1038/s41598-019-53766-2

**Published:** 2019-11-20

**Authors:** Zhuofa Chen, Dedong Han, Xing Zhang, Yi Wang

**Affiliations:** Institute of Microelectronics, Peking University, Beijing, 100871 China

**Keywords:** Electronic and spintronic devices, Electrical and electronic engineering, Electronic devices

## Abstract

In this paper, we investigated the performance of thin-film transistors (TFTs) with different channel configurations including single-active-layer (SAL) Sn-Zn-O (TZO), dual-active-layers (DAL) In-Sn-O (ITO)/TZO, and triple-active-layers (TAL) TZO/ITO/TZO. The TAL TFTs were found to combine the advantages of SAL TFTs (a low off-state current) and DAL TFTs (a high mobility and a low threshold voltage). The proposed TAL TFTs exhibit superior electrical performance, e.g. a high on-off state current ratio of 2 × 10^8^, a low threshold voltage of 0.63 V, a high field effect mobility of 128.6 cm^2^/Vs, and a low off-state current of 3.3 pA. The surface morphology and characteristics of the ITO and TZO films were investigated and the TZO film was found to be C-axis-aligned crystalline (CAAC). A simplified resistance model was deduced to explain the channel resistance of the proposed TFTs. At last, TAL TFTs with different channel lengths were also discussed to show the stability and the uniformity of our fabrication process. Owing to its low-processing temperature, superior electrical performance, and low cost, TFTs with the proposed TAL channel configuration are highly promising for flexible displays where the polymeric substrates are heat-sensitive and a low processing temperature is desirable.

## Introduction

Thin-film transistors (TFTs) has been widely applied for high-performance electronics applications such as Active Matrix Organic Light Emitting Diodes (AM-OLED). High-performance TFTs with a high mobility, a low threshold voltage, and a low swing slope can reduce the power consumption and enhance the quality of flat-panel display^1–5^. Therefore, various studies has be carried out to improve the electrical performance of TFTs, such as adopting different device structures^6^, using different channel materials, and optimizing the fabrication processes^7^.

TFTs fabricated by solution processing and inkjet printing have the advantage of low cost, while suffering from a low mobility and a high annealing temperature^8,9^. TFTs based on 2-dimentional (2D) materials such as graphene and Molybdenum disulfide (MoS_2_) have been widely investigated recently due to their excellent electrical properties^10,11^. However, 2D materials-based TFTs still have some challenges in large-scale fabrication of high quality devices, not compatible with modern Silicon-based microelectronic technologies. Zinc-oxide (ZnO) based TFTs have attracted considerable attention for their superior electrical and optical properties since last decade^2,3,12–14^. Among ZnO-based multicomponent oxide TFTs, In-Ga-Zn-O, Al-Zn-O, In-Zn-O, Zn-In-Sn-O TFTs had been proved to be attractive alternatives to conventional silicon-based TFTs in AMOLED due to their high mobility, low threshold voltage, fully transparency, and large-area applications^15–21^. While most of these work required a high processing or annealing temperature (above 300 °C). These thermal processes increase the manufacturing cost and limits their application in flexible display where a low processing temperature (<100 °C) is desirable^15,22,23^. Thus, alternative ZnO-based TFTs fabricated at a low temperature still need to be investigated. Sn-doped ZnO (TZO) has the advantages of high mobility and low temperature processing compatibility^24,25^. While the research of TZO TFTs received less attention and the device performance presented is undesirable. High-performance TZO TFTs fabricated at a low temperature are still of interest. Therefore, the goal of our research is to realize high-performance TZO TFTs at a low temperature.

The idea of adopting multi-stacked active-layer structures to improve the performance of TFTs has been previously investigated^26–30^. TFTs with LaTiO_3_/SrTiO_3_ heterostructure and ZnO-based ZnO/Zn_1−x_Mg_x_O heterostructure has been proved to exhibit a higher mobility than TFTs with conventional thin films and bulk materials^29,31,32^. Multi-stacked channel structures were also adopted in solution processed TFTs to improve the mobility of devices^26,27^. However, a systematic work to probe the performance of TZO TFTs with multi-stacked active-layer structure at a low processing temperature is still lacking. Previously, we reported improving the performance of TZO TFTs with various strategies such as adding oxygen during the deposition of TZO layers^33,34^, adopting DAL ITO/TZO TFTs^35,36^, and adjusting the thickness of the ITO/TZO active layer^37^. We demonstrated that TZO TFTs are promising switching devices for flat-panel applications. The DAL TFTs can effectively improve the mobility and reduce the threshold voltage. However, the DAL TFTs have a high off-state current due to the high carrier density in the ITO layer, leading to a higher power consumption. Therefore, we aim to optimize the channel structure of TFTs to reduce the off-state current and improve the on-off current ratio.

In this paper, we compared the performance of TFTs with different channel structures and demonstrated that high-performance TZO TFTs can be realized at a low temperature (80 °C) by adopting TAL stack for TFTs. Compared to TFTs with SAL or DAL channel configuration, the proposed TAL TZO/ITO/TZO TFTs exhibit a higher mobility and a lower threshold voltage. The quality of the TZO film and ITO films were characterized by AFM, SEM, and XRD. The stability and uniformity of our fabrication process is confirmed by the consistent performance of TAL TFTs with different channel lengths. A physical mechanism for the electrical improvement is also deduced. The proposed TAL TFTs are promising in various applications due to the superior performance, low-processing temperature, and low cost.

## Results

### Device structure and fabrication process

**Figure 1:**
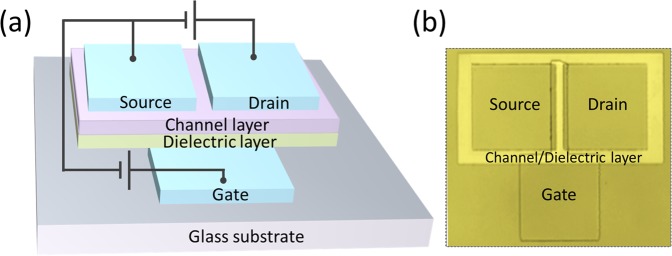
(**a**) Schematic illustration of the device structure. An inverted staggered structure was adopted in this research. The channel layer and the dielectric layer are patterned using the same mask. (**b**) An optical photo (top view) of a representative device in this paper.

**Figure 2:**
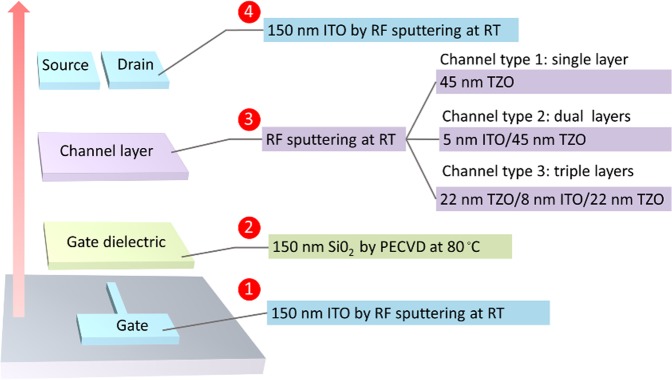
Fabrication process of the TFTs with three different channel configurations: channel type 1 (SAL), channel type 2 (DAL), and channel type 3 (TAL). The devices were fabricated from step 1 to step 4, successively.

A schematic of the device structure is shown in Fig. 1a. A bottom-gate TFT was fabricated on a glass substrate by standard photolithography and lift-off techniques, without any intentional substrate-heating process. All procedures were carried out below 80 °C. A top-view optical image of a representative device is shown in Fig. 1b. The device was fabricated using a 3 photo-masks process, as shown in Fig. 2. The detailed fabrication procedures are described in methods.

### Electrical measurements

**Figure 3:**
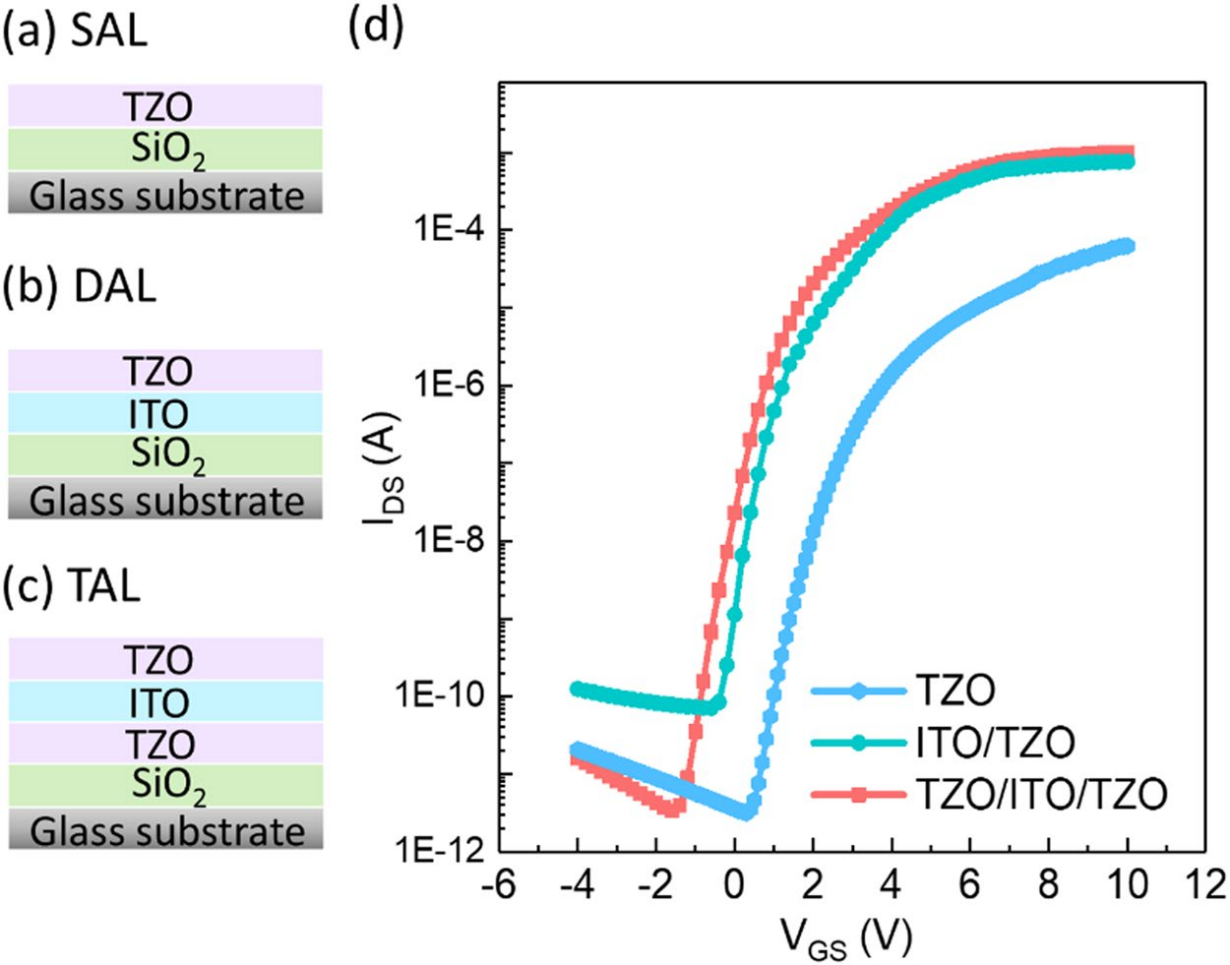
Schematic of (**a**) TZO single-active layer, (**b**) ITO/TZO dual-active layers, (**c**) TZO/ITO/TZO triple-active layers. (**d**) Representative transfer curves of TFTs with the three different channel configurations.

Figure 3a–c shows schematics of three different channel configurations: SAL, DAL, and TAL. Figure 3d shows the representative transfer curves of TFTs with three different channel configurations: TZO/ITO/TZO (TAL), ITO/TZO (DAL), and TZO (SAL). All the devices have the same channel dimension with a channel aspect ratio of 100 *μm*/20 *μm*. The drain to source voltage was biased at 5 V. Back-gate voltage was biased from −4V to 10 V. The transport measurements were carried out under ambient condition at room temperature. Figure 3d shows that the TAL TFTs have the best performance with a high on-off state current ratio (I_on_/I_off_) of ~2 × 10^8^ and a low V_th_ of ~0.6 V. Moreover, TAL TFTs has a high *μ*_*FET*_ of 128.6 cm^2^/Vs and a low I_off_ of 3.3 pA. The Swing Slope (*SS*) was calculated by the Eq. (1), while V_th_ and *μ*_*FET*_ were extracted by the Eq. (2). C_ox_ of 2.6 × 10^8^ F/cm^2^ was extracted from C-V curve of 100 K Hz^38^.

1 $$SS=\frac{\partial {V}_{GS}}{\partial (log{I}_{DS})}{/}_{{V}_{DS}=con}$$

2 $${I}_{DS}=\frac{W}{2L}{\mu }_{FET}{C}_{ox}({V}_{G}-{V}_{th}){V}_{DS}$$

**Figure 4:**
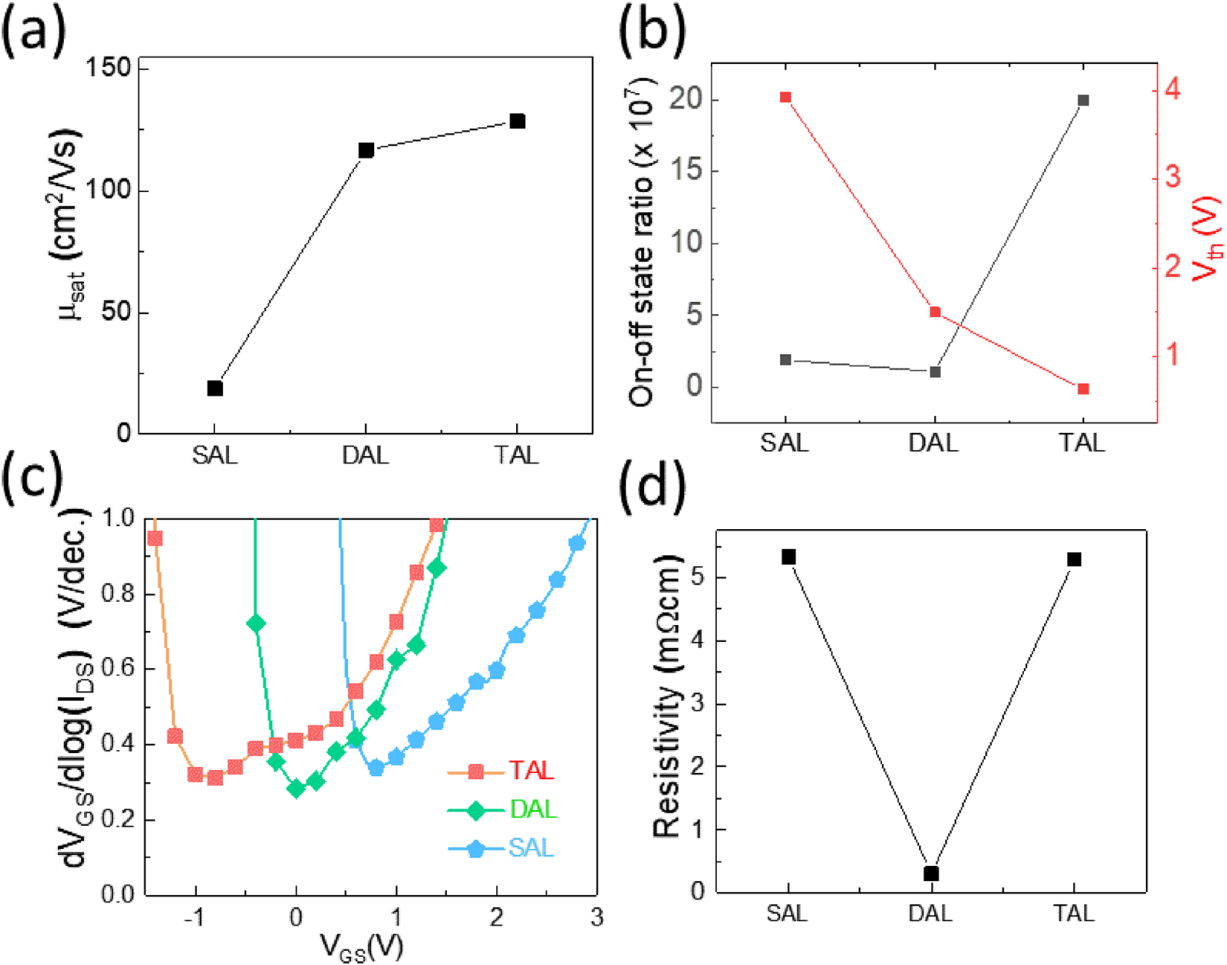
Comparison of the electrical properties of TFTs with different channel structures. (**a**) field effect mobility, (**b**) on-off state current ratio and threshold voltage, (**c**) subthreshold slope, and (**d**) channel resistivity.

Figure 4 compares the electrical properties of TFTs with three different channel configurations. Figure 4a compares the *μ*_*FET*_ of the devices. We can see that comparing to SAL TZO TFTs, the TFTs with TAL and DAL channel configurations have a much higher *μ*_*FET*_ (roughly 5 times higher). This high mobility is due to the good conductivity of the ITO layer in the channel^39^. Figure 4b compares the I_on_/I_off_ and V_th_ and shows that the TAL TFTs have the lowest V_th_ and the highest I_on_/I_off_. The DAL TFTs and SAL TFTs has similar I_on_/I_off_. After adding the ITO layer, both the I_on_ and I_off_ of the DAL TFTs are increased. Compared to SAL TFTs, DAL TFTs has the advantages of high *μ*_*FET*_ and low V_th_ while also suffering from a high I_off_, which may leads to higher power consumption in applications. The TAL TFTs combine the advantages of SAL TFTs (low I_off_) and DAL TFTs (high *μ*_*FET*_ and low V_th_). Figure 4c shows the variation of *SS* due to back gate voltage in TFTs with TAL, DAL, and SAL, respectively. All the TFTs have similar values of *SS* (~0.3 V/dec.). Figure 4d shows the channel resistivity of the SAL stack, DAL stack, and TAL stack, which was measured using 4-probe station. The SAL stack and TAL stack have roughly the same channel resistivity, ~20 times larger than that in DAL stack. This confirms the lower I_off_ in TAL and SAL TFTs while the higher I_on_ in DAL TFTs, shown in Fig. 3d. Extracted parameters were summarized in Table 1.

**Table 1:** Extracted parameters of the TFTs studied in this paper.

Channel stacks	Chanel width/Length	Thickness (nm)	μ_FET_ (cm^2^/V⋅s)	SS mV/dec.	V_th_ (V)	On-off Ratio
TZO	100/20	45	18.6	341	3.92	1.9 × 10^7^
ITO/TZO	100/20	5/45	116.7	282	1.5	1.1 × 10^7^
TZO/ITO/TZO	100/20	22/8/22	128.6	312	0.63	2 × 10^8^
TZO/ITO/TZO	100/80	22/8/22	120.8	353	0.89	1.0 × 10^8^
TZO/ITO/TZO	100/100	22/8/22	114.3	380	1.2	1.1 × 10^8^

These electrical results are originated from the different roles of each film in the channel. As the n-channel TZO TFTs operated on enhancement mode, most of the induced carriers go either into the deep localized states in the TZO layer or into the interface states when the gate bias voltage V_GS_ < 0 V. Only a very small fraction of electrons that are close to the front of TZO/SiO_2_ interface (interface near to the gate electrode) participate in channel conduction, resulting in a low I_off_. While as the V_GS_ increases, the channel conductivity increases rapidly due to charges accumulating in the TZO layer, yielding a suitable a high I_on_. The TZO channel controls the charge conductance to get a high I_on_/I_off_ and a suitable V_th_. While for DAL ITO/TZO TFTs, the high mobility electron gas formed in the high density interface of the ITO/TZO heterostructure, leading to a higher mobility and I_on._ The TZO layer provides a suitable V_th_ due to its controlling ability in the charge conductance. Compared to TZO conducting layer, the thin ITO layer of the DAL ITO/TZO channel provides a higher carrier concentration, therefore maximizing the charge accumulation and yielding a high *μ*_*FET*_, while suffering a high off-state current^40^.

For TAL TFTs with TZO/ITO/TZO channel strucutre, there are three different interfaces that affects the electrical characteristics of the device: two ITO/TZO interfaces (above and below the ITO layer) in the channel stack, and the TZO/SiO_2_ interface. The high-density TZO/ITO interface and the high charge density ITO layer form electron gas and account for the high mobility and high saturation current. The TZO/SiO_2_ interface may not have high trap density as the swing slope in this device is low. During the turn on and off operation, less electrons are trapped in TZO/SiO_2_ interface. The TZO layer has low charge density and maintain low I_off_, thus the device has good controllability on the channel conductance.

**Figure 5:**
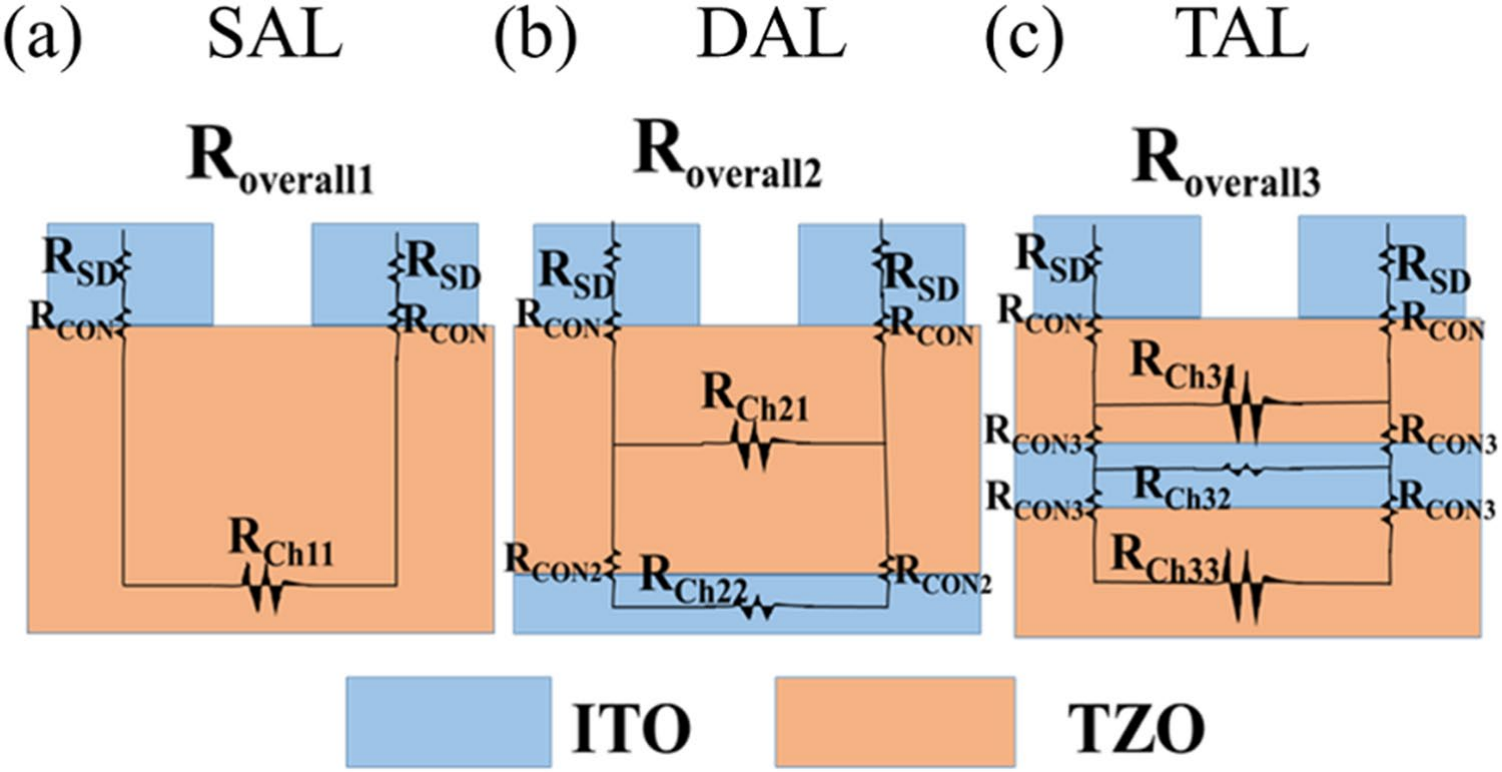
Schematic cross-sectional view of the overall resistance in (**a**) SAL TZO TFTs, (**b**) DAL ITO/TZO TFTs and (**c**) TAL TZO/ITO/TZO TFTs. R_SD_ is the resistance in the source and drain electrode, R_CON_, R_CON2_, R_CON3_ are the interface resistances between interface and R_CH11_, R_CH21_, R_CH22_, R_CH31_, R_CH32_, R_CH33_ are the resistances in the active layers.

From the resistance point of view, the ITO layer reduces the channel resistance of ITO/TZO TFTs while encapsulating the ITO layer between two TZO layers can increases the channel resistance. The schematic illustration of the three different channel configurations is shown in Fig. 5. Compared to the SAL TZO TFTs, the high carrier density in the ITO layer, leads to smaller channel layer resistance R_ch22_ and R_ch32_ (shown in Fig. 5b,c)^41^, resulting in a smaller overall resistance of ITO/TZO TFTs (R_overall2_) despite small contact resistance R_con_ and R_con2_. Using the Eq. (3):

3 $${I}_{off}=\frac{{V}_{DS}}{{R}_{overall}}$$

While for the TAL, thinner TZO layer has lower carrier density, yielding larger channel resistances R_ch31_ and R_ch33,_ the series resistance R_con3_ also adds to R_overall3_ (shown in Fig. 5(c))^37,42^. Thus, R_overall3_ is larger than the R_overall2_. This can be confirmed by the resistivity shown in Fig. 4d. Therefore, the TAL TFTs have lower I_off_ than the DAL TFTs.

### Output characteristics and device stability

**Figure 6:**
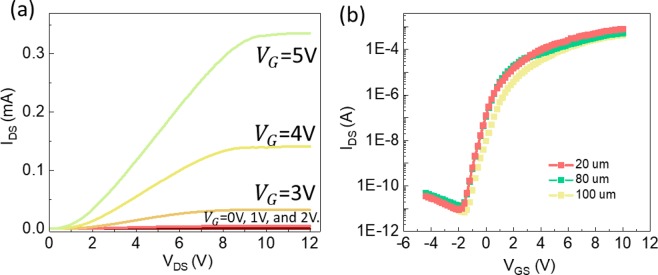
(**a**) Output characteristics of the TAL TFTs. (**b**) Representative transfer characteristics of TAL TFTs with channel length of 20 µm, 80 µm, and 100 µm.

Figure 6a shows the output characteristics of the TAL TFTs. The TAL TFTs work on enhancement mode and the back-gate voltage was set from 0 V to 5 V with a step of 1 V. The drain and source voltage scans from 0 V to 12 V. The drain current is raised rapidly within 1 V between drain and source and a clear saturation region can be observed. This demonstrates the good switch controlling ability (switch from off-state to on-state rapidly) of the device. Figure 6a shows that the saturation current exceeds 300 µA at a low back-gate voltage of 5 V. This indicates good current driving ability in the TAL TFTs. However, nonlinear correlation between the V_DS_ and the I_DS_ was also observed for V_DS_ < 1 V. This may be due to the parasitic resistance induced by trap states near source and drain regions, leading to the current crowding phenomenon. Part of the drain voltage may drop on the parasitic resistance^43^. Due to the limitation of our setup, all the electrical characteristic measurements were performed under ambient condition. Oxygen may be adsorbed on the top of the channel and form a depletion layer. This may also lead to current crowding phenomenon. More work can be done to improve the quality of the contact interface but that’s out of the scope of this paper. Moreover, later work can optimize the device structure by adding an insulating layer on top of the channel to prevent this problem.

To investigate the stability and the uniformity of our fabrication process. TAL TFTs with different channel lengths are also fabricated and measured. Figure 6b shows the representative transfer characteristics of TAL TFTs with three different channel length 20 µm, 80 µm, and 100 µm. The related parameters were extracted and shown in Table 1. All the devices have comparable mobility higher than 100 cm^2^/Vs and high on-off state current ratio higher than 10^8^. This indicates our fabrication process is stable and uniform.

### Material surface morphology and transparency

**Figure 7:**
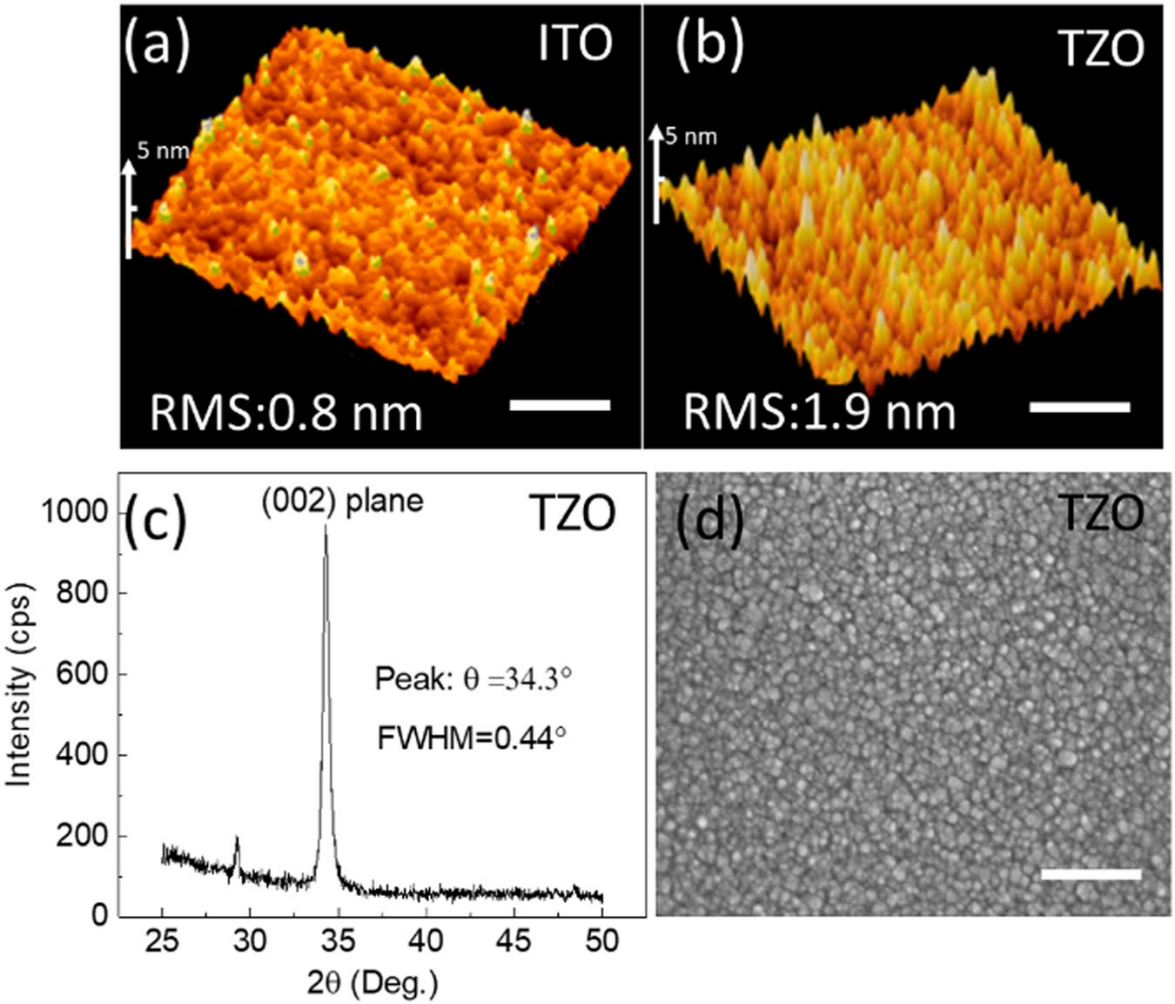
Surface characterization off the ITO and TZO films. (**a**) AFM image of the ITO film. (**b**) AFM image of the TZO film. (**c**) XRD diffraction pattern of the TZO film. (**d**) SEM micrograph of the TZO film. The scale bar for AFM images and SEM images is 200 µm.

Figure 7a,b show the AFM surface morphology of the ITO and TZO film, respectively. The RMS is 0.8 nm and 1.9 nm, respectively. The smooth surface of the ITO film indicates better conductance of the film while the TZO film has a granular surface morphology with a larger surface roughness. The X-ray diffraction in Fig. 7c has one prominent peak at 34.3°, indicating Sn atoms successfully replace Zn sites in the lattice and form C-axis-aligned crystalline (CAAC)^44–46^. The average grain size of the TZO film is estimated to be 17.1 nm using the Scherer formula, this can also be confirmed by the SEM image shown in Fig. 7d.

## Discussion

For SAL TZO TFTs, oxygen was intentionally added during the RF sputtering process of the TZO film to reduce oxygen vacancy in the material, leading to reduction the hole density in the channel, which can reduce the off-state current and improve the swing slope of the device^34^. This can explain the low off-state current in SAL TZO TFTs. For the DAL ITO/TZO TFTs, ITO layer with a high carrier density was introduced to form channel layer. The high carrier density improves the mobility and the on-state current. Though the DAL ITO/TZO TFTs have superior performance including a high mobility, a low V_th_, and a low *SS*, the high off-state current will lead to a high power consumption in real applications. The TAL channel configuration proposed in this paper has lower off-state current and still maintain a high mobility, can effectively solve this problem. Note that the thickness of the channel stack can also affect the performance of the devices. We have previously reported TZO TFTs and ITO/TZO TFTs with various TZO film thickness and ITO film thickness^36,37^. The thickness of the channel layers of the SAL TFTs and DAL TFTs in this research has been optimized. Thus, we can eliminate the effect of channel thickness when comparing the performance of devices with three different channel configurations. A more systematic work on optimizing the thickness of TAL stacks can be done to further improve the performance of the TAL TFTs. But this would not affect our comparison of the three channel configurations and demonstration of the superior performance of the TAL TFTs.

## Conclusions

In this paper, we compared the electrical properties of TFTs with three different channel configurations including SAL, DAL, and TAL. Compared to SAL TFTs, DAL TFTs has a higher mobility and a lower *SS* due to the high carrier density from the ITO layer. While DAL TFTs suffer from a high off-state current, which leads to a higher power consumption in real application. The TAL TFTs were proposed to solve this problem. The proposed TAL TFTs combine the advantages of both SAL TFTs and DAL TFTs and exhibit superior electrical performance such as a high on-off state current ratio of 2 × 10^8^, a low V_th_ of 0.63 V, a high *μ*_*FET*_ of 128.6 cm^2^/Vs, and a low off-state current of 3.3 pA. Owing to its advantages of low-processing temperature and superior electrical performance, TFTs with the proposed TAL channel configuration are highly promising for oxide semiconductor TFTs manufacturing and have application in flexible displays where the use of heat-sensitive polymeric substrates is desirable. Thus, this investigation is very crucial for commercial applications.

## Methods

### Device fabrication

The fabrication procedures are described as follows: (1) A gate electrode was patterned and a 150-nm thick ITO film was deposited by radio frequency (RF) magnetron sputtering at room temperature (RT) in Ar (pressure: 1.2 Pa and power: 70 W). (2) A 150-nm thick SiO_2_ was grown using plasma-enhanced chemical vapor deposition (PECVD) with a mixture of SiH_4_ and N_2_0 (ratio 65:130) at 80 °C. (3) Channel layers were deposited by RF sputtering at room temperature in Ar/O_2_ mixture (flow rate ratio 100/8) with a power of 70 W. The target adopted for sputtering was a ceramic target with a mass ratio of ZnO: SnO_2_ = 97: 3. In this paper, TFTs with three different channel configurations were fabricated. (a) Single-active-layer TFTs (SAL TFTs) with single TZO layer (channel type 1 in Fig. 2), a 45-nm thick TZO was growth by RF sputtering. (b) Dual-active-layer TFTs (DAL TFTs) with ITO/TZO stack (channel type 2 in Fig. 2), a 5-nm thick ITO was first deposited and followed by depositing a 45-nm thick TZO. (c) Triple-active-layer TFTs (TAL TFTs) with TZO/ITO/TZO stack (channel type 3 in Fig. 2), 22-nm thick TZO, 5-nm ITO, and 22-nm TZO were deposited sequentially by RF sputtering. (4) After patterning the source and drain electrodes, a 150-nm thick ITO film was RF sputtered and lifted to form the source and drain electrodes.

### Device measurement and materials characterizations

The surface morphology of the TZO films and ITOs films were evaluated by atomic force microscopy (AFM) and scanning electron microscope (SEM). The structure of the TZO film was analyzed by X-ray powder diffraction (XRD). The channel resistivity was obtained from four-probe station. The transport properties of the TFTs were characterized by a semiconductor parameter analyzer (Agilent 4156C). The resistivity of the stacks was measured using the 4-probe station.

## Data Availability

The datasets generated during and/or analyzed during the current study are available from the corresponding author on reasonable request
